# Neurocognitive deficits and socioeconomic risk factors among children and adolescents living with HIV in sub-Saharan Africa: a systematic review

**DOI:** 10.1186/s13034-022-00465-y

**Published:** 2022-04-27

**Authors:** Otsetswe Musindo, Lydiah Krabbendam, Joan Mutahi, Miguel Pérez García, Paul Bangirana, Manasi Kumar

**Affiliations:** 1 Department of Clinical, Neuro and Developmental Psychology, Amsterdam Public Health Research Institute, Vrije Universiteit Amsterdam, Amsterdam, The Netherlands; 2 Department of Psychiatry, University of Nairobi, Nairobi, Kenya; 3 Facultad de Psicología, Universidad de Granada, Granada, Spain; 4 Department of Psychiatry, Makerere University, Kampala, Uganda; 5 Department of Psychiatry , University of Nairobi to Brain and Mind Institute Aga Khan University , Nairobi, Kenya

**Keywords:** Children and adolescents living with HIV, Neurocognitive deficits, Socioeconomic factors, Systematic review

## Abstract

**Introduction:**

Children and adolescents living with HIV (C/ALHIV) are at a risk for significant neurocognitive deficits. There is limited literature that addresses the role of socioeconomic factors in neurocognitive deficits among CALHIV in Sub Saharan Africa (SSA), as it is very difficult to establish this causal relationship. Our systematic review was guided by the biodevelopmental framework that assumes that foundations of health and adversity affect later development and life outcomes. This systematic review aims to assess available evidence on the relationship between neurocognitive deficits and socioeconomic factors among HIV children and adolescents in SSA region.

**Method:**

Using a pre-determined search strategy, we searched electronic databases including PubMed, web of Science and EBSCOhost (CINAHL and MEDLINE). Peer-reviewed publications that address neurocognitive deficits, psychosocial and socioeconomic risk factors among children and adolescents living with HIV in SSA were included in review.

**Results:**

Out of 640 articles, 17 studies from SSA met the inclusion criteria. Four studies reported no significant differences in the neurocognitive measures comparing children and adolescents with HIV infection to those uninfected. However, 10 studies suggest that C/ALHIV scored significantly low in general intellectual functions as compared to their uninfected peers. C/ALHIV were found to have substantial deficits in specific cognitive domains such as sequential processing, simultaneous processing, and learning. In addition, deficits in visuo-spatial processing, visual memory and semantic fluency were mentioned. Socioeconomic factors such as lower socioeconomic status (income, education and occupation), child orphanhood status and under-nutrition were linked with neurocognitive deficits.

**Conclusion:**

Our findings suggest that CALHIV presented with poorer neurocognitive outcomes when compared to other populations which were associated with specific socioeconomic factors.

## Introduction

Children and adolescents living with HIV (C/ALHIV) are at high risk of developing neurocognitive deficits. Sub Saharan Africa (SSA) has the highest number of HIV infections of any region in the world, with an estimate of 460,000 (1–45) newly infected with HIV. In the era of increased uptake of Highly Active Antiretroviral Therapy (HAART), the child and adolescent survival rates have improved. However, current research indicates that neurocognitive deficits and associated morbidities persist [1–3].

High prevalence of poor neurocognitive functioning among C/ALHIV has been consistently reported in studies in SSA [4–7] with neurocognitive deficits as early as infancy [8, 9] in preschool [10] and in school aged children [11]. C/ALHIV are likely to show cognitive deficits in specific domains such as attention, processing speed, language, motor skills, learning and memory, visual spatial abilities and executive functioning [12–14]. Socioeconomic factors and psychosocial outcomes play an important role in cognitive outcomes of C/ALHIV [2, 11, 15]. This has led to a debate whether these cognitive deficitsstem from HIV or rather, are a result of socioeconomic factors affecting young people in particular, such as home environment [16].

Previous meta-analyses and systematic review have addressed the following areas; i) the extent of cognitive impairment in perinatally HIV infected children and adolescents compared to HIV negative controls and specific domains commonly affected [17], ii) neuropsychological tools focusing on norming and adaptation in SSA [18] and iii) interventions for children with neurocognitive impairments in resourceslimited settings [19]. There is a paucity of data that addresses children and adolescents (10–24 years) compared to studies done among younger ages, even though neurocognitive deficits may persists in adolescence and adulthood [16]. The following question is the focus of this review: Is there an impact of socioeconomic factors, psychosocial outcomes and HIV biomedical factors on neurocognitive functioning in C/ALHIV in SSA region?

**Fig. 1:**
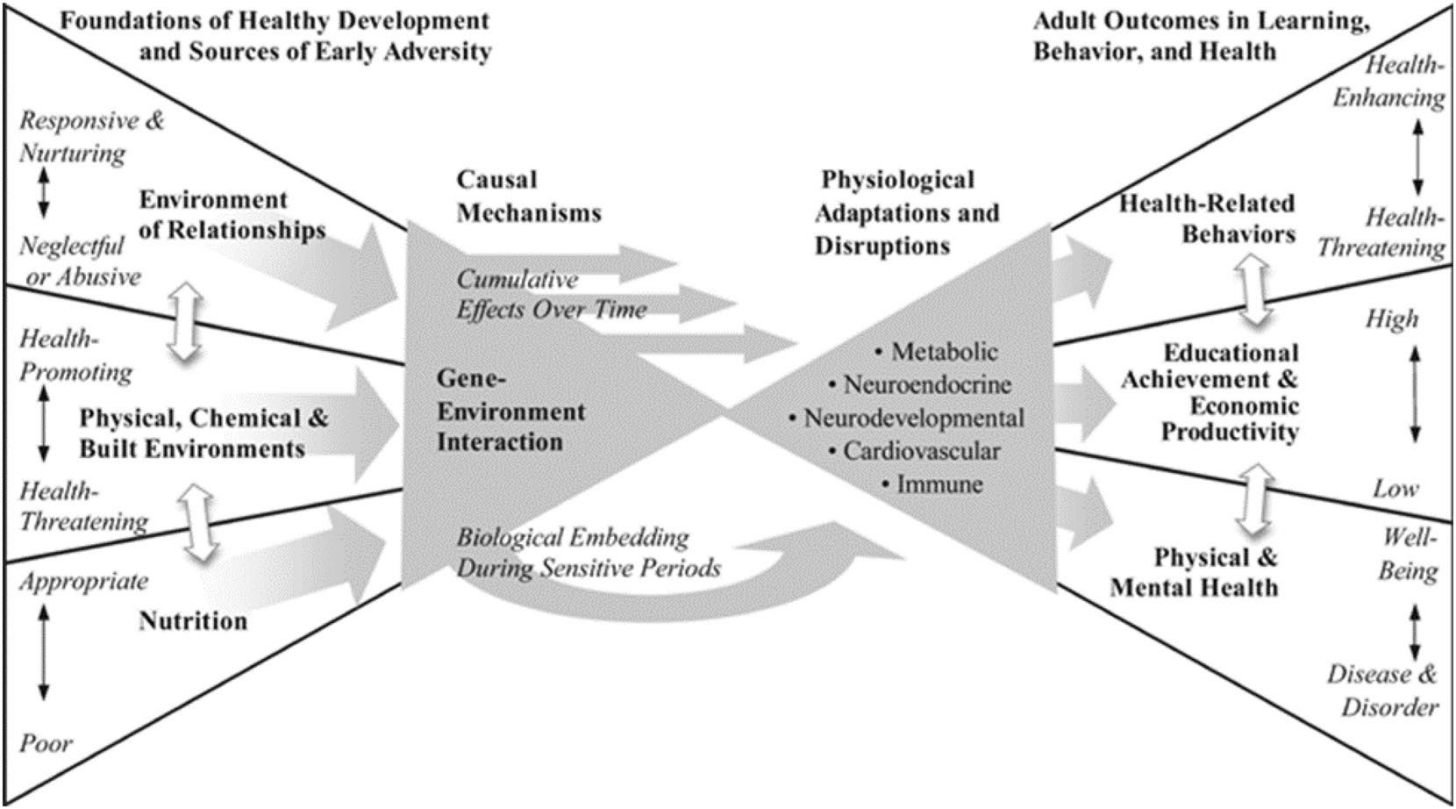
Bio-developmental framework by Shonkoff [Source: Shonkoff, 20]

Our systematic review was guided by the biodevelopmental framework developed by Shonkoff [20] which assumes that foundations of health and adversity affect later development and life outcomes. These outcomes are in turn influenced by the cumulative burden of risk factors and the buffering effects of protective factors within the environment of an individual. Positive early experiences are vital for healthy development and adaptation. In extending this framework, Shonkoff and Phillips [21] state that families and communities play a vital role in providing supportive relationships and positive learning experiences that young children need for healthy development. Therefore, this model is important in understanding HIV related challenges faced by C/ALHIV as well as expected clinical, cognitive and mental health outcomes. Illustrated in Fig. 1 are the pathways modelling the key factors faced by C/ALHIV in SSA.

## Methods

The PRISMA (Preferred Reporting Items for Systematic reviews and Meta-Analyses guidelines [22] was used to guide the systematic review and checklist by Joanna Briggs Institute (JBI)) [23]. We started the search on the 30th May 2020. After completion of the search on the 2nd April 2021, quality assessment and writing of the review commenced. The review itself was completed on 12th August 2021.

### Search strategy

We devised a search strategy which included electronic databases of the following sources: PubMed, EBSCOhost (CINAHL and Medline), PsycINFO and [Forward AND backwards snowballing] = (hand-search/control studies). When conducting the searches, search terms were combined using Boolean terms “AND” “OR”. The articles were searched using the following keywords (See Table 1.).

**Table 1:** Search strategy

Keywords	Synonyms
“Neurocognitive deficit*”	‘Neurodevelopment/al’ OR ‘neurocognitive’ OR ‘cognitive’ OR ‘cognitive function’ OR neurocognitive function OR ‘neurodevelopmental’ OR Neurocognitive impairment OR Neurocognitive status OR Neurocognitive dysfunction*
“Children and adolescents”	Adolescen* OR Teen* OR Youth OR Young adult* OR Young people OR Young person OR Young men OR Young women OR Youngster* OR Juvenile* OR Child* OR “School-aged child*”
HIV infected	HIV-infected OR “living with HIV” OR HIV OR AIDS OR HIV/AIDS OR Human immunodeficiency virus OR Human immuno-deficiency virus OR Acquired immunodeficiency OR Antiretroviral OR ARV*
“Socioeconomic factor*”	Standard* of Living OR Living Standard* OR Social Class OR Economic Status OR Educational Status OR Level of education OR Educational attainment OR Employment OR Income OR Family OR Community safety OR Social support OR welfare OR Nutrition levels OR Healthcare OR Medical indigency OR age
Sub-Saharan Africa	Sub Saharan Africa OR Sub Sahara Africa OR Angola OR Benin OR Botswana OR Burkina Faso OR Burundi OR Cameroon OR Cape Verde OR Central African Republic OR Chad OR Comoros OR Democratic Republic of the Congo OR Djibouti OR Equatorial Guinea OR Eritrea OR Ethiopia OR Gabon OR The Gambia OR Ghana OR Guinea OR Guinea-Bissau OR Ivory Coast OR Kenya OR Lesotho OR Liberia OR Madagascar OR Malawi OR Mali OR Mauritania OR Mauritius OR Mozambique OR Namibia OR Niger OR Nigeria OR Republic of the Congo OR Rwanda OR Sao Tome and Principe OR Senegal OR Seychelles OR Sierra Leone OR Somalia OR South Sudan OR Sudan OR Swaziland OR Eswatini OR Tanzania OR Togo OR Uganda OR Zambia OR Zimbabwe

In terms of PICOS: Children and adolescents living with HIV (Population) Socioeconomic risk factors (Intervention) HIV Unexposed Uninfected, HIV exposed Uninfected comparably high poverty sample (Comparative/ Control intervention) Neurocognitive (Outcomes) and Cross-sectional studies, case control, cohort studies and clinical trials (study designs).

### Inclusion criteria

Studies were included when they met the following criteria: (a) children and adolescents living with HIV between the ages 6 to 18 years. (b) Neuropsychological test measure was used OR cognitive outcome reported in the study. (c) Study conducted in SSA. (d) Socioeconomic and psychosocial risk factors were assessed using socio demographics questionnaire or psychological tools.

### Exclusion criteria

This review did not include studies that were not published in the English language. The time restriction was not included due to limited number studies on neurocognitive functioning and socioeconomic challenges in SSA.

### Selection process

**Fig. 2:**
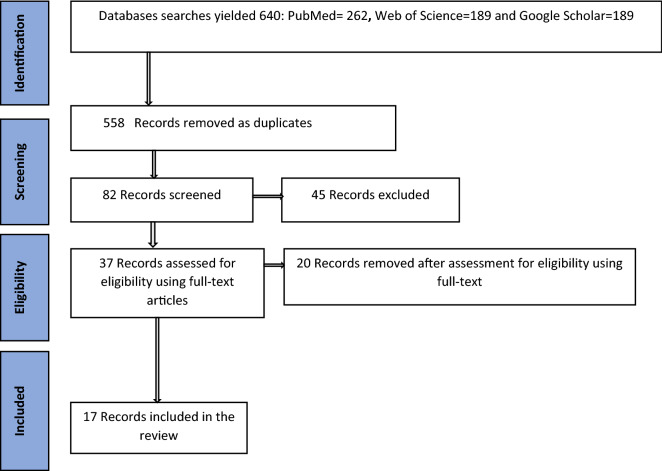
Flow diagram of the study selection process

The search identified 640 articles (see Fig. 2). The initial screening was done by two independent reviews (JM and OM) based on the title, abstract with reference to inclusion and exclusion criteria. Each reviewer screened titles and then abstracts to select the articles that met the inclusion criteria. Those articles that did not meet the inclusion and exclusion criteria were excluded. Full texts of 37 articles were reviewed and lead to the elimination of 20 articles that did not meet the inclusion criteria. After completed screening, two reviewers (JM and OM) met to seek consensus on the selected articles. As a result, 17 articles initially met the inclusion criteria and were eligible for the review (see Fig. 2). The next stage involved capturing all articles that were relevant on the first screening using a structured Microsoft excel spreadsheet developed by the study team. Articles were downloaded for in-depth review and two reviewers (JM and OM) examined the full texts again to make a final decision regarding inclusion according to the eligibility criteria. They examined the articles independently and extracted the most relevant information that was included in a spreadsheet. Basic information such as the title, year, author, country, study design, sample, neuropsychological tool, general cognition and specific domains and socioeconomic risk factors were captured. Disagreements between the 2 reviewers regarding the inclusion or exclusion of particular studies were settled by consultation with a third reviewer (MK).

### Quality of study methodology

The methodological guidance for systematic review developed by Joanna Briggs Institute (JBI) was used to assess the quality of the selected studies [23]. All selected studies based on the inclusion criteria were subjected to appraisal by at least 2 reviewers (OM. JM). The purpose of was to assess the methodological quality of a study and to determine the possibility of bias in its design, conduct and analysis.

### Main outcome(s)

The review outcome of interest is to assess neurocognitive, neurodevelopment and cognitive functioning domains and to review evidence on whether socioeconomic risk factors are associated with neurocognitive functioning among C/ALHIV. In addition, whether there is an interrelationship that the evidence points to which can inform intervention development or intervention implementation.

### Strategy for data synthesis

We conducted a narrative synthesis for all the included studies and all data extracted from the articles were presented narratively in text and summary tables. Similarities and differences in study designs, populations and the outcome measures were highlighted and patterns in the data identified. To report our results accurately, we used the Guidance on the Conduct of Narrative Synthesis in Systematic Reviews developed by Popay et al. [24].

## Results

Our systematic review yielded seventeen eligible studies. The PRISMA flowchart below provides the details of the study selection process. Six studies originated from Uganda, four studies from Nigeria and one study each from Cameroon, South Africa, Kenya and Zimbabwe. Studies were predominantly based in hospitals, clinics or other healthcare facilities. The sample size ranged from 12 to 611 and participants ranged from 6 to 15 years. The Kaufman Assessment Battery for Children second edition (KABC-II) [25] and Raven’s Progressive Matrices (RPM) [26] tests were the most commonly used neurocognitive assessments. Results are presented according to, (a) the presence of neurocognitive deficits (b) specific domains (c) HIV biomedical outcomes, (d) socioeconomic factors and (e) psychosocial outcomes.

### Methodological quality of included reviews

The appraisal results for the included studies are outlined in Table 2. In the critical appraisal checklist, all studies included in the review obtained above 80% “yes” response. They all used validated tools to measure neurocognitive functioning of C/ALHIV in SSA. Musindo et al. did not include a control group [6] and two of the studies [12, 27] had inadequate sample size. Sixteen of the studies clearly described the sampling method, Boivin et al. [28] did not provide adequate sampling procedure information. Overall, all studies included in the review reported measurement of the outcome in a reliable way.

**Table 2:** Quality of studies

Source	Q1	Q2	Q3	Q4	Q5	Q6	Q7	Q8	Q9
Bangeda et al.,2006	Y	Y	N	Y	Y	Y	Y	Y	Y
Boivin et al., 2010(a)	Y	U	Y	Y	Y	Y	Y	Y	Y
Boivin et al.,2010(b)	Y	Y	Y	Y	Y	Y	N	Y	Y
Hoare et al., 2012	Y	Y	N	Y	Y	Y	Y	Y	Y
Ruel et.al., 2012	Y	Y	Y	Y	Y	Y	Y	Y	Y
Boyede et.al., 2013	Y	Y	Y	Y	Y	Y	Y	Y	Y
Boyede et al., 2013	Y	Y	Y	Y	Y	Y	Y	Y	Y
Boyede et al., 2013	Y	Y	Y	Y	Y	Y	Y	Y	Y
Kandawasvika et al	Y	Y	Y	Y	Y	Y	Y	Y	Y
Boivin et al., 2016	Y	Y	Y	Y	Y	Y	Y	Y	Y
Iloh et. al., 2017	Y	Y	Y	Y	Y	Y	Y	Y	Y
Brahmbhatt et al., 2017	Y	N	Y	Y	Y	Y	Y	Y	Y
Musindo et.al., 2018	Y	Y	Y	Y	Y	N	Y	Y	Y
Boivin et al., 2018	Y	Y	Y	Y	Y	Y	Y	Y	Y
Debeaudrap et al., 2018	Y	Y	Y	Y	Y	Y	Y	Y	Y
Familiar et al., 2019	Y	Y	Y	Y	Y	Y	Y	Y	Y
Boivin et al., 2020	Y	Y	Y	Y	Y	Y	Y	Y	Y

^*^Y = *Yes, N* = *NO, U* = *unclear, NA* = *Not applicable*

1) Was the sample frame appropriate to address the target population?

2) Were study participants sampled in an appropriate way?

3) Was the sample size adequate?

4) Were the study subjects and the setting described in detail?

5) Was the data analysis conducted with sufficient coverage of the identified sample?

6) Were valid methods used for the identification of the condition?

7) Was the condition measured in a standard, reliable way for all participants?

8) Was there appropriate statistical analysis

9) Was the response rate adequate, and if not, was the low response rate managed appropriately?

The Joanna Briggs Institute. The Joanna Briggs Institute Critical Appraisal tools for use in JBI Systematic Reviews—Checklist for Prevalence Studies. Crit Apprais Checkl Preval Stud. 2017;7

### General intellectual functioning (general cognition)

Table 3 summarizes studies reporting general cognition, specific domains and socioeconomic risk factors of C/ALHIV in SSA region. Five different neuropsychological assessments RPM, KABC-II, Wechsler Abbreviated Scale of Intelligence—Second Edition, (WASI-II) and McCarthy Scales of Children’s Abilities (MSCA) were the included studies. These measures assess general cognition and are standardized for the use in children and adolescents [1, 4, 11, 28–31]. The evidence on general intellectual functioning among C/ALHIV varied across the studies. 10 studies found cognitive deficits among HIV infected children and adolescents as compared to their negative controls (both exposed and unexposed). However, 3 studies reported that there were no significant differences in terms of general intellectual functioning outcomes for the HIV infected and control groups [12, 27, 29, 32].

**Table 3:** Summary of studies reporting general cognition and specific domains and psychosocial aspects of C/ALHIV in SSA

**Author**	**Year & country**	**Sample (n)**	**age**	**setting**	**Study design**	**Neuropsychological tools**	**General Cognition and specific domains**	**Socioeconomic risk factors**
Bangeda et al.	2006, Uganda	107, 28 HIV + , 42-, 37c	6–12 years	hospital	Cohort	K-ABC, WRAT-3	HIV + , no significant cognitive difference No information about specific cognitive domains	showed significantly more evidence of acute malnutrition
Boivin et al.	2010a, Uganda	102 clinical group	6–12 years	Hospital	Cross sectional	KABC-II, TOVA, BOTS, HOME,	Children with HIV subtype A performed more poorly than those with HIV subtype D on all measures Performed poorly on sequential processing (p = 0.01), simultaneous processing(p = 0.005), Learning (p = 0.03)	None
Boivin et al.	2010b, Uganda	60 PHIV 23 on HAART	6–16 years	Hospital	Cross sectional	Captain's Log CCRT, KABC-II, Cogstate, SES physical quality of home environment checklist	Sequential processing p = 0.01, simultaneous p = 0.02, learning, p = 0.05	None
Hoare et al.	2012, South Africa	12 HIV + , 12 HIV-	8–12 years	Clinics	Cross sectional	WASI-II	performed significantly worse than controls on all of the measures deficits in visuo-spatial processing, visual memory and semantic fluency	None
Ruel et al.	2012, Uganda	93 HIV + , 106 HIV-	6–12 years	Hospital	Cross sectional	KABC-II TOVA BOT-2	HIV + children performed significantly worse than HIV-uninfected children Deficits in sequential processing and planning/reasoning as compared with HIV- HIV + with CD4 cell counts of > 350 cells/μL demonstrate significant cognitive and motor deficits Higher HIV RNA level was associated with poor performance in simultaneous processing (coefficient, − 4.5; P = .015) Impairment among those WHO stages 1 and 2 reported in sequential processing and planning	None
Boyede et al.	2013 a, Nigeria	(138) 69 HIV + 69 HIV -	6–15 years	Hospital	Cross sectional	RPM	RPM cognitive scores for HIV positive children are lower than those of HIV negative No information about specific cognitive domains	younger age(p = 0.01), Low level of maternal education (p = 0.001) and low SES was associated with poor cognitive outcomes
Boyede et al.	2013b, Nigeria	69 HIV + 69 HIV-	6–15 years	Hospital	Cross sectional	RPM	Had significantly lower cognitive scores compared with HIV negative children No information about specific cognitive domains	None
Boyede et al.	2013c, Nigeria	69 HIV + , 39 on HAART 30 not on HAART	6–15 years	Hospital	Cross sectional	RPM	RPM scores tended to be lower with worsening WHO clinical stage No information about specific cognitive domains	None
Kandawasvika et al.	2015, Zimbabwe	n = 306 32 HIV infected, 121 exposed uninfected 153 unexposed uninfected	6–8 years	clinics	Cross sectional	MSCA	No difference in general cognitive function Deficits in perceptual performance in HIV infected group	Unemployed caregivers, undernutrition, child orphanhood were associated with impaired cognitive performance in univariate analysis
Boivin et al.	2016, Uganda	159	6–12 years	Hospital	Randomized Controlled Trial (Group 1 CCRT n = 53, Group 2 Limited CCRT n = 52, Group 3 Control n = 54)	Captain's Log CCRT, KABC-II, CogStateBruininks/ Oseretsky test; BRIEF, CBCL, TOVA	At baseline, performed poorly on simultaneous processing (p = .035), learning (p = .047), knowledge (p = .001), NVI (p = .001) The CCRT group had significantly greater gains through 3 months of follow-up compared to passive controls on overall KABC-II mental processing index, planning, and knowledge The limited CCRT group performed better than controls on learning	None
Iloh et al.	2017, Nigeria	200 (100 HIV + and 100 HIV-)	6–15 years	Hospital	Cross sectional	RPM	lower cognitive functioning was noted among HIV positive compared with HIV negative peers No information about specific cognitive domains	all children with mother with no formal education performed below average. SES (p ¼ 0.028) and immunologic stage (0.015) had significant negative effect on RPM scores of HIV-positive children
Brahmbhatt et al.	2017, Uganda	370, 204 HUU, 26 PHEU, 140 PHIV	7–14 years	Clinics	Cross sectional	KABC-II	No significant differences in the neurocognitive measures between PHIV and HUU PHIV had an impairment in simultaneous processing, learning and knowledge skills compared with PHUU and PHEU at baseline	increases in both age standardized weight and height resulted in significant improvement of sequential and simultaneous processing skills
Musindo et al.	2018, Kenya	90 HIV +	8–15 years	Hospital	Cross sectional	KABC-II, HEADS_ED	60% scored below 2SD High prevalence was seen in Simultaneous processing, planning and Nonverbal index	education and activities and peer support was associated with poor neurocognitive outcomes
Boivin et al.	2018 South Africa, Zimbabwe, Malawi, Uganda	611 246 HIV + , 183 HEU, 182 HUU	5–11	Clinics	observational multicentre longitudinal study	KABC-II TOVA BOT-2 BRIEF SES MICS4	HIV + children performed poorly than both HUU and HEU on the composite scores (mental processing index) deficits in sequential processing (working memory) learning, delayed recall, planning, simultaneous, non-verbal index as compare to negative controls	Area of residence, height for age, paternal level of education were associated with low cognitive scores
Debeaudrap et al.	2018, Cameroon	338 127 HIV-infected, 101 HEU, 110 HUU	4–9 years	Hospital	Cross sectional	SDQ KABC-II	HIV-infected children performed significantly worse than HUU children on MPI scores HEU children also had significantly lower MPI, NVI, learning and planning scores than HUU children	Mother’s education and vital status, caregiver depression and anxiety scores and household income HIV-infected children had higher SDQ scores than HUU children indicating that they experienced more behavioural difficulties
Familiar et al.	2019, Zimbabwe, South Africa, Uganda and Malawi	611 183 HEU 182 HUU 246 HIV-I	5–11	Clinics		Hopkins Symptom Checklist (HSCL) KABC-II TOVA BOT-2 BRIEF	MPI scores were significantly lower among HIV + children compared with HEU and HUU children No information about specific cognitive domains	Caregiver depressive symptomatology was not associated with other assessed KABC-II (MPI) scores
Boivin et al.	2020, South Africa, Zimbabwe, Malawi, Uganda	611 183 HEU 182 HUU 246 HIV-I	5–11	clinics	Observational multicentre longitudinal study	KABC-II TOVA BOT-2 BRIEF	The HIV + cohort performed significantly worse than the HEU and HUU cohorts for all KABC-II Deficits in simultaneous processing, sequential processing, learning, planning and delayed recall as compared to negative controls	Higher SES index scores were predictive of better KABC scores

*RPM* Ravens Progressive Matrices, *KABC-II* Kaufman Assessment Battery for children- Second edition, *WASI-II* Wechsler Abbreviated Scale of Intelligence—Second Edition, *MSCA*- McCarthy Scales of Children `s Abilities, *AWMA* Automated Working Memory Assessment

*PHIV* Perinatally HIV Infected, *PHEU *Perinatally HIV Exposed but Uninfected, *HUU *HIV Unexposed and Uninfected

### Specific Cognitive Domains

Seven studies reported significant differences between C/ALHIV and HIV-uninfected controls (both exposed and unexposed), in specific cognitive domains. C/ALHIV performed poorly on simultaneous processing (visual-spatial processing and problem solving), learning (immediate and delayed memory) [5, 28, 29, 33] and sequential processing (memory) [5, 33, 34]. In Zimbabwe, Kandawasvika et al. [32] found that C/ALHIV scored lowest in the perceptual performance domain. In South Africa, Hoare et al. [27] noted lower scores on visuo-spatial processing, visual memory, semantic fluency, motor functioning, processing speed, executive functioning.

### Socioeconomic risk factors

Three studies [4, 31, 35] that investigated neurocognitive functioning among C/ALHIV in Nigeria and Cameroon reported that maternal education was associated with neurocognitive outcomes. Boyede et al. [4] found that children of mothers with primary or no education (*p* = 0.001) were almost three times more likely to have below average RPM scores compared with children of mothers with secondary or tertiary education [1]. In Zimbabwe, orphanhood and caregiver unemployment status (odd ratio of 2.1 (95% CI 1.03–3.4) was associated with low score on MSCA [32]. Caregiver depression and anxiety scores were associated with lower scores on KABC-II [35] while Familiar et al. [36] did not find any association. In addition, Iloh et al. [31] indicated that that socio-economic class (*p* = 0.028) had significant negative effect on RPM scores of HIV positive children. Undernutrition, stunting and wasting were associated with neurocognitive functioning [12, 32].

### Psychosocial Outcomes

Three studies [6, 35, 37] assessed psychosocial outcomes by using child behavior checklist (CBCL), Strength and Difficulty Questionnaire (SDQ) and Home, Education, Activities and peers, Drugs and alcohol, Suicidality, Emotions and behaviours, and Discharge resources (HEADS-ED). A Cameroonian study found that C/ALHIV experienced more behavioral difficulties and this was indicated by higher SDQ scores [35] indicating that they experienced more behavioral difficulties. In addition, SDQ scores were associated with the cognitive scores such as mental processing index, nonverbal index, simultaneous, planning and sequential, learning for children with score ≥ 1 SD compared with the other. Using the HEADS-ED, a study by Musindo et al. [6] found that children and adolescents who are experiencing considerable problems at school and also the greater the dissatisfaction a caregiver expressed about school performance, the lower the scores on mental processing index (overall cognition). There was no association between CBCL behavior/symptom ratings and cognitive scores [37].

### HIV Biomedical outcomes

Three studies in Uganda and Nigeria [11, 31, 34] found poor cognitive performance in relation to WHO clinical stages of HIV infection among CALHIV. Significantly lower scores in the RPM among 6–11 years on HAART were associated with worsening WHO clinical stages (*p* = 0.03) and those not on HAART (*p* = 0.001). In addition, Iloh et al. [31] found that C/ALHIV with severe suppression (≤ 200 CD4 count cells per millimeter) (0.015) performed poorly on RPM scores. Those with high levels of RNA (Viral load) was associated with poor performance on simultaneous processing while with CD4 cell counts of > 350 cells/μL demonstrate significant cognitive and motor deficits that correlate with HIV plasma RNA level (viral load) [34].

## Discussion

We identified 17 studies that highlighted neurocognitive and socioeconomic risk factor faced by C/ALHIV in SSA. C/ALHIV presented with poorer neurocognitive outcomes when compared to other populations which were associated with socioeconomic factors [4, 29, 31, 32, 34, 37].The majority of studies used KABC and RPM to measure neurocognitive outcomes. Van Wyhe et al. [38] confirm that KABC-II can be used widely across different countries due to its strong psychometric properties and cultural appropriateness. The RPM has been validated among the Nigerian children [39], however, there is a controversy about its cultural equivalence [40].Therefore, these measures provided critical information on neurocognitive functioning. However, there is a need to develop local instruments that can provide precise understanding of neurocognitive functioning.

Our findings indicate that there is conflicting evidence of cognitive performance among C/ALHIV. Some of the studies found that C/ALHIV perform more poorly in neurodevelopmental assessments than uninfected controls [4], although in some, no significant differences between groups [12, 27, 29, 32]. Smith et al. [41] explains that differences in the neurocognitive outcomes may be attributed to the HIV biomarkers (Viral load, CD4 count and WHO clinical stage). The lack of routine screening for this population makes it impossible to provide accurate detection and better understanding of neurocognitive deficits for further intervention in SSA. There is need for culturally sensitive tool or adaptation of tools from those initially developed in the West [32], however, developing a new tool is complex and expensive especially in resource-limited settings.

C/ALHIV have shown substantial deficits in cognitive domains such as sequential processing, simultaneous processing and learning [28]. Therefore, C/ALHIV have deficits in both general intellectual functioning to severe deficits in specific cognitive domains and “when assessing the cognitive functioning of children with HIV, it is imperative to examine both global (e.g. intelligence quotient) and specific (e.g. processing speed, visual-spatial) domains of functioning [42].” Our findings suggest an association between poor cognitive performance and World Health Organization clinical stages of HIV infection among C/ALHIV [11, 31, 34] ongoing cognitive decline regardless of being on medication and the undetectable viral load [4]. This is supported by Ravindran et al. [2] and Jeremy et al. [43], however, Laughton et al. [44] found that infants who received ART before 3 months of age had better neurodevelopmental scores compared to infants for whom ART was delayed. Long term use of HAART in young children have been linked to improved neurocognitive outcomes [10].

Our findings suggests that low socioeconomic background contribute to poor neurocognitive outcomes in children infected with HIV [1, 31, 32]. This finding concurs with Ravindran, Rani and Priya [2], Abubakar et al. [45] and Coscia et al. [16]. Boivin et al. [5] concluded that younger children, who come from poor rural areas tend to be stunted, lacked preschool education and that parents’ educational level is important. SES influences availability of cognitive stimulation in the home, this includes such books, computers and many more. This suggests that educated mothers provide a cognitively stimulating environment that is conducive to cognitive improvement in children and adolescents.

The bio-developmental framework concurs that a safe environment and consistent presence of stable caring adults are critical for social and cognitive development that they can use throughout their lives [20]. The caregiver is the most critical person and Shonkoff [20] describes caregiver in the home or institutional setting as the people who relate closely with children such as individual and groups within a community, in school, and in health facilities. This is clear indication that there is need to strengthen home and family environment by focusing on empowering caregivers of C/ALHIV and training them on ways to improve psychosocial and neurocognitive function. A previous study by Boivin et al. [8] showed that neurocognitive deficits among HIV infected in SSA are more likely to be pathophysiology of HIV infection but also poor nutrition and home environment. However, our systematic review failed to assess other factors which are highlighted by the biodevelopmental framework such as exposure to substance and secondary infections in utero, prematurity and birth weight, early experiences of trauma which influence neurocognitive outcomes [20]. We do think that these areas warrant further exploration and the need to address the psychosocial and home environment context of children living with HIV.

## Limitations of the study

One limitation of this systematic review is the significant heterogeneity in study designs and reported outcomes, which limited comparisons between the results and conclusions across the articles. Our systematic review did not include other factors which are highlighted by the bio-developmental framework such as exposure to substance and secondary infections in utero, prematurity and birth weight, early experiences of trauma which influence neurocognitive outcomes. We do think that these areas warrant further exploration and the need to address the psychosocial and home environment context of children living with HIV.

## Conclusion

This systematic review has important implications for clinical and public health interventions for C/ALHIV. It highlights some of the psychosocial factors, HIV biomedical outcomes and most importantly socioeconomic risk factors that are associated with poor neurocognitive functioning. Given the fact that HIV continues to create a significant burden in the region, there is need for further research and tailored neurocognitive interventions that focus on socioeconomic risk factors. In addition, there is a need to locally developed and validated neurocognitive tests that are culturally appropriate for use in this population in the SSA region.

## Data Availability

All data generated or analyzed during this study are included in this published article.

## Abbreviations

C/ALHIV: Children and adolescents living with HIV
HAART: Highly Active Antiretroviral Therapy
SSA: Sub Saharan Africa
KABC-II: Kaufman Assessment Battery for children-Second Edition
RPM: Raven’s Progressive Matrices
WASI-II: Wechsler Abbreviated Scale of Intelligence—Second Edition
MSCA: McCarthy Scales of Children’s Abilities
SDQ: Strength and Difficulty Questionnaire
